# Research on the correlation between activating transcription factor 3 expression in the human coronary artery and atherosclerotic plaque stability

**DOI:** 10.1186/s12872-021-02161-9

**Published:** 2021-07-28

**Authors:** J. Peng, C. Y. Le, B. Xia, J. W. Wang, J. J. Liu, Z. Li, Q. J. Zhang, Q. Zhang, J. Wang, C. W. Wan

**Affiliations:** Department of Forensic Medicine, Guizhou Medical University, Guiyang, 550000 Guizhou China

**Keywords:** Atherosclerosis, ATF3, Plaque stability, Inflammatory reaction, Coronary heart disease

## Abstract

**Background:**

Activating transcription factor 3 (ATF3) is an early response gene that is activated in response to atherosclerotic stimulation and may be an important factor in inhibiting the progression of atherosclerosis. In this study, we directly measured the expression of ATF3 and inflammatory factors in human coronary atherosclerotic plaques to examine the relationship between ATF3 expression, inflammation and structural stability in human coronary atherosclerotic plaques.

**Methods:**

A total of 68 coronary artery specimens were collected from the autopsy group, including 36 cases of sudden death from coronary heart disease (SCD group) and 32 cases of acute death caused by mechanical injury with coronary atherosclerosis (CHD group). Twenty-two patients who had no coronary heart disease were collected as the control group (Con group). The histological structure of the coronary artery was observed under a light microscope after routine HE staining, and the intimal and lesion thicknesses, thickness of the fibrous cap, thickness of necrosis core, degree of lumen stenosis were assessed by image analysis software. Western blotting and immunohistochemistry were used to measure the expression and distribution of ATF3, inflammatory factors (CD45, IL-1β, TNF-α) and matrix metalloproteinase-9 (MMP-9) and vascular cell adhesion molecule 1 (VCAM1) in the coronary artery. The Pearson correlation coefficient was used to analyse the correlation between ATF3 protein expression and inflammatory factors and between ATF3 protein expression and structure-related indexes in the lesion group.

**Results:**

Compared with those in the control group, the intima and necrotic core in the coronary artery were thickened, the fibrous cap became thin and the degree of vascular stenosis was increased in the lesion group, while the intima and necrotic core became thicker and the fibrous cap became thinner in the SCD group than in the CHD group (*P* < 0.05). There was no or low expression of ATF3, inflammatory factors, VCAM1 and MMP-9 in the control group, and the expression of inflammatory factors, VCAM1 and MMP-9 in the SCD group was higher than that in CHD group, while the expression of ATF3 in the SCD group was significantly lower than that in CHD group (*P* < 0.05). In the lesion group, the expression of ATF3 was negatively correlated with intimal and necrotic focus thickness, positively correlated with fibrous cap thickness (*P* < 0.01), and negatively correlated with inflammatory factors, VCAM1 and MMP-9 (*P* < 0.01).

**Conclusions:**

The expression of ATF3 may be related to the progression and stability of atherosclerotic plaques, and may affect the structural stability of atherosclerotic plaques by regulating the inflammatory response, thus participating in the regulation of atherosclerotic progression.

**Supplementary Information:**

The online version contains supplementary material available at 10.1186/s12872-021-02161-9.

## Background

Coronary heart disease (CHD) is the leading cause of death due to noncommunicable diseases worldwide, accounting for 16% of the total global deaths [1]. Atherosclerosis (AS), the pathological basis of CHD, is considered to be a complex chronic inflammatory disease that occurs in the walls of blood vessels [2]. Many kinds of inflammatory cells, including mononuclear macrophages, are involved in the occurrence and development of AS [3]. However, the aetiology and pathogenesis of CHD are still unclear in clinical medicine and forensic medicine research.

In recent years, dysfunctional cell signal transduction and gene expression have been suggested to be key molecular mechanisms of cardiovascular disease [3–5]. Transcription factors directly affect gene expression and play key roles in regulating cell function and disease development [6]. Activating transcription factor 3 (ATF3), a member of the ATF/cAMP response element binding (CREB) protein family, is important transcription factor [7, 8]. ATF3 expression level is low in quiescent cells but can be induced in response to various stimuli, such as cytokines and chemokines [9–11]. Studies have shown that ATF3 gene dysfunction is related to many pathophysiological reactions, such as inflammation, signal transmission, apoptosis, oxidative stress and endoplasmic reticulum stress, and ATF3 is involved in a variety of pathophysiological changes, such as extracellular matrix dysfunction, smooth muscle cell proliferation and migration, and foam cell formation, in the development of atherosclerosis [12]. However, whether ATF3 is a promoter or inhibitor has not been determined, and its role and mechanism in atherosclerosis are not clear. Most of the previous researches were based on serological or animal experiments, and there have been few reports on human atherosclerosis. In this study, coronary arteries from autopsy cases were used to directly examine the degree of inflammation and the expression of ATF3 in human coronary artery lesions and to explore the relationship between the expression of ATF3, the level of inflammation in atherosclerotic plaques and the structural stability of atherosclerotic plaques. To provide a richer experimental basis for the diagnosis or treatment of coronary heart disease.

## Methods

### Case selection

Autopsy cases were selected from the Forensic Judicial Expertise Centre of Guizhou Medical University from January 2017 to December 2019. The principles outlined in the Declaration of Helsinki and the Rules for Autopsy issued by the Ministry of Health of the People’s Republic of China were followed. It had obtained the informed consent from donor or next of kin of the deceased and was approved by the Ethics Review Committee of Guizhou Medical University (approval number: 201801). Sixty-eight coronary artery tissue specimens were included in the lesion group: there were 36 cases of sudden death of coronary heart disease caused by arrhythmia or myocardial infarction, accompanied by plaque rupture and intra-plaque haemorrhage (SCD group) and 32 cases of death caused by traffic accident, electric shock, etc., and coronary artery associated with atherosclerosis (CHD group). Twenty-two cases of acute death caused by traffic accident, electric shock, etc., and no coronary heart disease were used as the control group (Con group).

The following inclusion criteria were applied to the disease group. (1) The cadaver was cryopreserved within 6 h of death, and the autopsy was performed within 48 h of death. (2) The presence of coronary artery AS was confirmed by the naked eye and histological examination. Coronary artery intimal thickening, lumen narrowing, atherosclerotic plaque formation, atherosclerotic plaque rupture, intraplaque haemorrhage and aneurysm formation were observed in the thickened coronary artery intima, with or without any secondary lesions such as atherosclerotic plaque rupture, intraplaque hemorrhage, aneurysm formation, etc. (3) In CHD group, the deceased died quickly because of traffic accidents, electric shocks, etc. (4) In the SCD group, systematic autopsy and routine toxicological examination were performed within 24 h from the onset of coronary heart disease symptoms to death, excluding the possibility of poisoning or other diseases (Additional file 1).

The inclusion criteria of the control group was autopsy at the same time as the lesion group, no coronary artery disease as confirmed by the naked eye and histological examination. And the cause of death in control group was caused by traffic accidents, electric shocks, etc. The exclusion criteria were as follows: (1) tissue autolysis or unclear structure; (2) rheumatic heart disease, cardiomyopathy, myocarditis and other heart disease; and (3) multiple systemic inflammatory infections or multiple organ dysfunction.

According to the nano-exclusion standard, the left anterior descending coronary artery without any lesion was selected as the control group. The experimental group specimens were selected as the locations at which stenosis of the anterior descending coronary artery was the heaviest as observed by the naked eye, and the selected tissue was fixed with 4% formaldehyde. Part of the tissue was frozen at − 80 °C for protein extraction and analysis.

### Observation of coronary artery structure

The coronary artery tissue in all cases were fixed with 4% paraformaldehyde for 72 h. The cross section of the blood vessel was embedded in paraffin, 4-μm sections were prepared and stained with conventional HE, and an EasyScan digital section scanning and application system (McAudi Industrial Group Co., Ltd., China) was used to observe the morphology and structure of the coronary artery. IPP 6.0 image analysis software was used to detect the related morphological indexes[13]: (1) intimal and lesion thicknesses: From the free edge of the endocardial cavity surface to the vertical distance of the internal elastic membrane, the thickest and thinnest thickness of the intima was measured, and another straight line was perpendicular to the two test lines. The mean of the test line and the average thickness of the intima were calculated. (2) thickness of the fibrous cap: The fibrous cap on the surface of atherosclerotic lesions of coronary arteries was assessed in the experimental group. The thickness of the fibrous cap on both sides of the cap and at the thickest intimal was measured, and the average value was obtained. (3)Thickness of necrosis core: The vertical distance from the proximal rim to the proximal margin, the largest and smallest necrosis lesions in atherosclerosis were evaluated, and another test line was drawn perpendicular to the test line, with three lines in total. The mean value was the average thickness of the necrotic lesions, while necrosis was not evaluated. (4) Degree of lumen stenosis: The ratio of intimal area to the sum of intima and lumen area was assessed. The increase of intimal and lesion thicknesses and degree of lumen stenosis is related to the progression of atherosclerosis. The thinning of the fibrous cap and the enlargement of necrosis core are related to the plaque instability of atherosclerosis.

### Immunohistochemical staining

The expression and distribution of target proteins in coronary artery lesions were analysed by immunohistochemistry (IHC). There were 12 cases in each group. The coronary arteries were sectioned in paraffin and baked at 60 °C for 1 h and hydrated with concentration gradient after being dewaxed with xylene. Endogenous peroxidase was blocked with 3% H_2_O_2_, hyperbaric heat repair antigen was performed for 4 min, and the samples were blocked with goat serum and then incubated with antibodies against ATF3 (1:150, ^#^ab254268, Abcam, UK), CD45 (1:200, ^#^20103–1-AP, Proteintech, China), IL-1 β (1:150, ^#^abs131179, Absin, China), TNF-α (1:100, ^#^YM3478, Immunoway, China), MMP-9 (1:50, ^#^10375-2-AP, Proteintech, China), and VCAM1 (1:200, ^#^abs119895, Absin, China). PBS was selected as negative control and incubated with the sections overnight in wet box at 4 °C. On the next day, the primary antibody was washed away with PBS, and horseradish peroxidase-labelled sheep anti-rabbit IgG secondary antibody was added. After being incubated for 30 min at 37 °C, DAB was used for colour development, and the samples were stained with haematoxylin, cleared with xylene and sealed with neutral gum. The positive expression of the target protein was observed under a microscope, and was visualized as a brown colour. Three visual fields were selected and pictures were taken at 400 × magnification. The mean optical density (MOD) of the positive signal was measured by IPP6.0 software to evaluate the degree of target protein expression (MOD = positive expression/total measured area). The result is the average value of 3 repeated measurements.

### Western blotting

Protein expression in the coronary artery was analysed by Western blotting. Vascular tissue was stored at − 80 °C, the homogenate was prepared, and the protein concentration was determined by ultraviolet spectrophotometry. SDS gel electrophoresis (10%) was performed, the proteins were transferred to a PVDF membrane, and the membrane was sealed with 5% skim milk for 2 h. According to the molecular size of the target protein (ATF3, 1:300; CD45, 1:1000; IL-1β, 1:1000; TNF-α, 1:1500; MMP-9, 1:600; VCAM1, 1:1000), the corresponding PVDF membranes were incubated overnight on a shaker at 4 °C. After the membranes were washed, HRP-conjugated goat ant-rabbit IgG (1:5000, Absin, China) was added and incubated at room temperature for 1 h. Chemiluminescent HRP substrate (Millipore, USA) was used for colour development on a chemiluminescence imaging system. β-actin was used as the internal reference, the grey value of the band was analysed and measured by ImageJ, and the mean value of three measurements was taken as the result.

### Statistical analysis

The data were analysed by SPSS 22.0 statistical software, and normal distribution and homogeneity of variance were tested. The measurement data are expressed as the mean ± SD. The mean of the homogeneity of variance was compared by single factor analysis of variance (LSD), and data with uneven variances were analysed by the Games-Howell test. The chi-square test was used to compare the counting data between the two groups, and correlations were analysed by the Pearson correlation coefficient test. The difference was statistically significant at *P* < 0.05 and *P* < 0.01. The number of case experiments is indicated in each figure legend.

## Results

### Basic case information

According to the nano-arrangement standard, 90 coronary artery specimens were collected. There were 22 cases in the control group, including 14 males and 8 females, aged 20–78 years old, with an average age of 35.14 ± 13.63 years. There were 32 cases in the CHD group, including 14 males and 18 females, aged 30–50 years old, with an average age of 38.56 ± 4.40 years. There were 36 cases of sudden death from coronary heart disease, including 19 males and 17 females, aged 47–74 years old, with an average age of 57.58 ± 7.18 years. Comparing the baseline data among the groups, there was no significant sex difference between the groups (*P* > 0.05). There was a significant difference in age among the groups (*P* < 0.05) (Table 1) (Additional file 1).

**Table 1:** Baseline case information

Group	Sex (male/female)	Sum	Constituent ratio (%)	Age (year)
Con	14/8	22	24.44	35.14 ± 13.63
CHD	14/18	32	35.56	38.56 ± 4.40^a^
SCD	19/17	36	40.00	57.58 ± 7.18^ab^
Total	–	90	100.00	–
*χ*^2^ or *F*	2.074	–	–	43.438
*P*	0.355	–	–	0.000

^a^*P* < 0.01 versus Con

^b^*P* < 0.01 versus CHD

### Morphological observations of the coronary artery

**Fig. 1:**
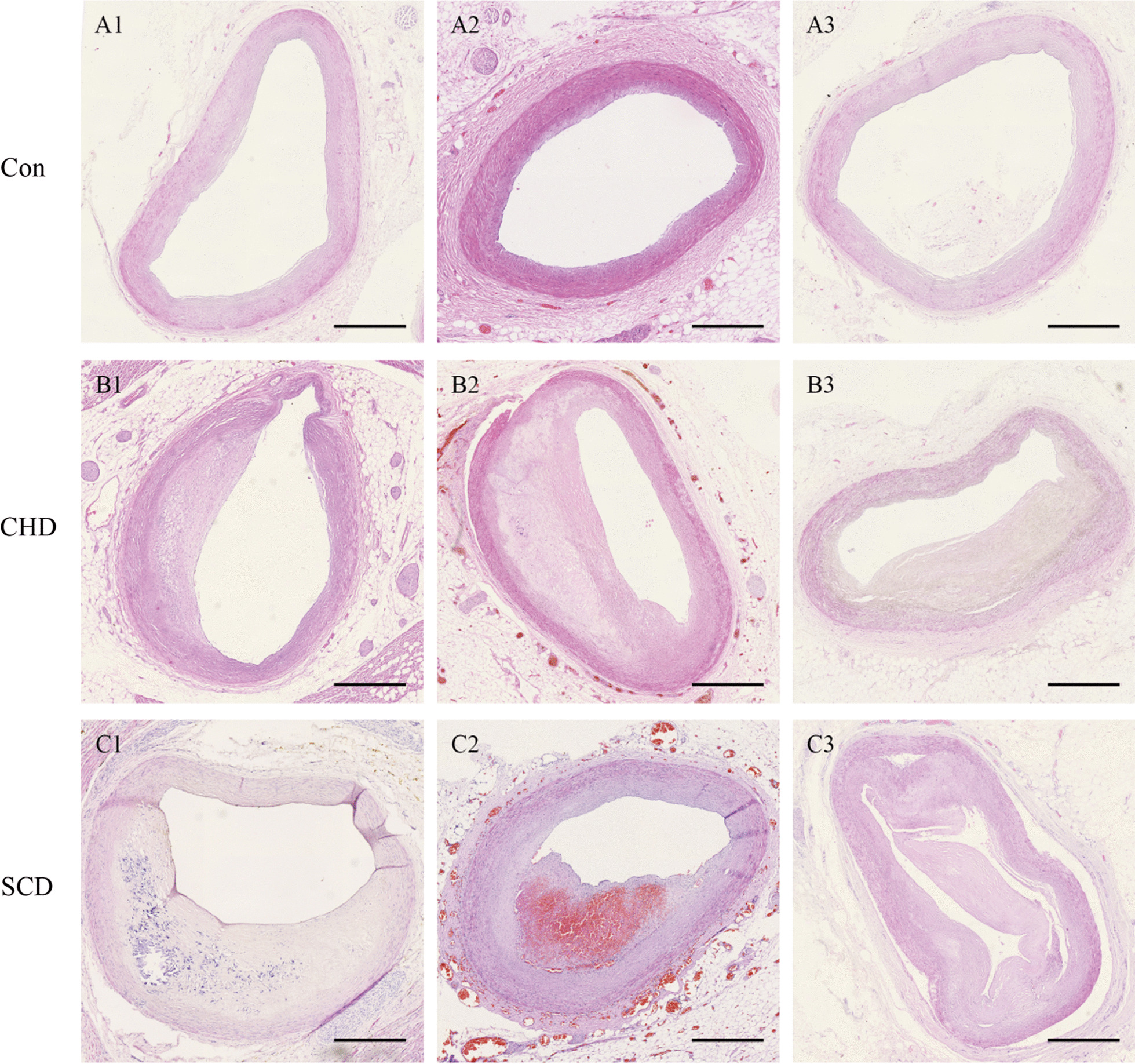
Microscopic changes in coronary atherosclerosis. **A1–A3** Control group, Con. **B1–B3** Coronary heart disease group, CHD. **C1–C3** Sudden death due to coronary heart disease with intraplaque bleeding and rupture group, SCD. (HE staining, scale bar = 1 mm)

The histopathological staining results showed that the endothelial cells of the coronary artery were smooth and intact in the control group. In CHD group, the intima of the coronary artery was adaptively thickened, typical fibrous caps were observed under the intima, and a small number of foam cells were seen under the fibers. In SCD group, the formation of necrotic core was seen under the intima, the fibrous cap covering the necrotic core became thinner or ruptured, and a large number of cholesterol crystals or intra-plaque hemorrhage could be seen in the necrotic focus (Fig. 1).

Image analysis showed that coronary intimal thickness and necrotic focus thickness in the CHD or SCD group were higher than those in the control group, the thickness of the fibrous cap and the degree of lumen stenosis increased in the lesion group (*P* < 0.05). Compared with those of the CHD group, the thickness of the coronary intima, the thickness of the necrotic focus and the degree of stenosis in the SCD group were increased, and the thickness of the fibrous cap was reduced (*P* < 0.05) (Table 2).

**Table 2:** Comparison of morphological indexes of coronary artery lesions (mean ± SD)

Grading	Cases (*n*)	Intimal and lesion thicknesses (mm)	Necrotic core thickness (mm)	Fiber cap thickness (mm)	Degree of lumen stenosis (%)
Control	22	1.79 ± 0.70	0.00 ± 0.00	0.00 ± 0.00	14.21 ± 5.93
CHD	32	6.17 ± 1.07^a^	0.00 ± 0.00	3.02 ± 0.78^a^	52.17 ± 13.29^a^
SCD	36	10.10 ± 2.40^ab^	3.07 ± 1.77^ab^	0.83 ± 0.29^ab^	63.44 ± 14.29^ab^
*F*	–	168.719	81.116	274.478	111.942
*P*	–	0.000	0.000	0.000	0.000

^a^*P* < 0.05 versus control

^b^*P* < 0.05 versus CHD

### ATF3 protein expression in coronary atherosclerotic lesions

**Fig. 2:**
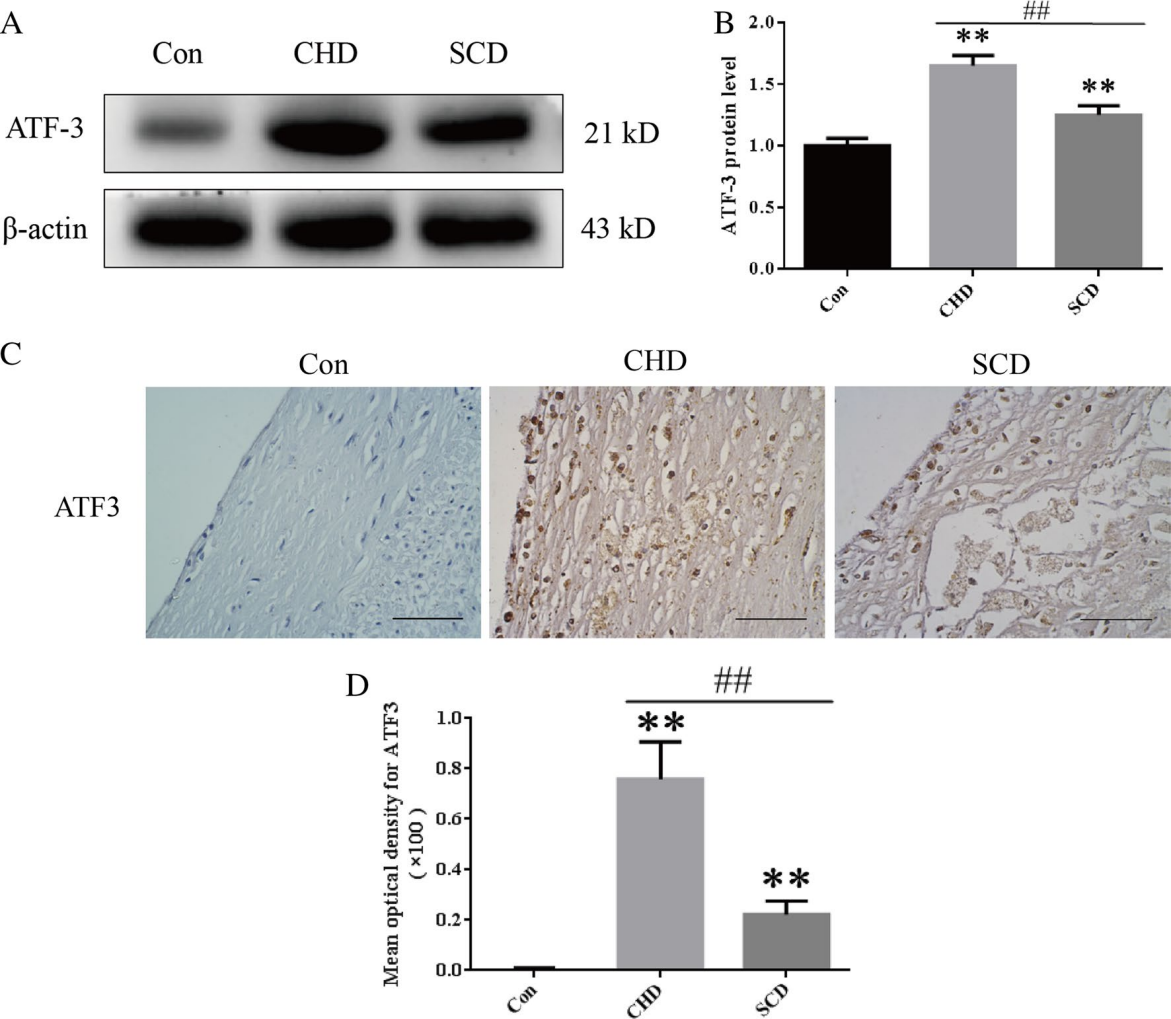
ATF3 expression levels in the coronary artery. **A, B** Representative Western blot images and quantitative analysis of ATF3 expression levels. **C, D** Representative immunohistochemical images and quantitative analysis of ATF3 expression in each group (scale bar = 50 μm). All experiments were repeated at least three times. The data are expressed as the mean ± SD (n = 12 per group). **P < 0.01 versus the control group; ##*P* < 0.01 versus the CHD group

The Western blot results showed that the expression of ATF3 in coronary artery tissue in the lesion group was higher than that in the control group, and the expression of ATF3 in the SCD group was lower than that in the CHD group (Fig. 2A, B, P < 0.01). The immunohistochemical results showed that there was no positive expression of ATF3 in the control group, but ATF3 was expressed in the cytoplasm and nucleus of foam cells in the shoulder area and bottom of the atherosclerotic focus in the disease group, as indicated by yellow or brown staining (Fig. 2C). The optical density was measured by IPP6.0 and showed that the MOD in the disease group was higher than that in the control group, while the MOD in the SCD group was significantly lower than that in the CHD group (Fig. 2D, P < 0.01) (Additional file 2).

### Expression of CD45, IL-1β and TNF-α in the coronary artery

**Fig. 3:**
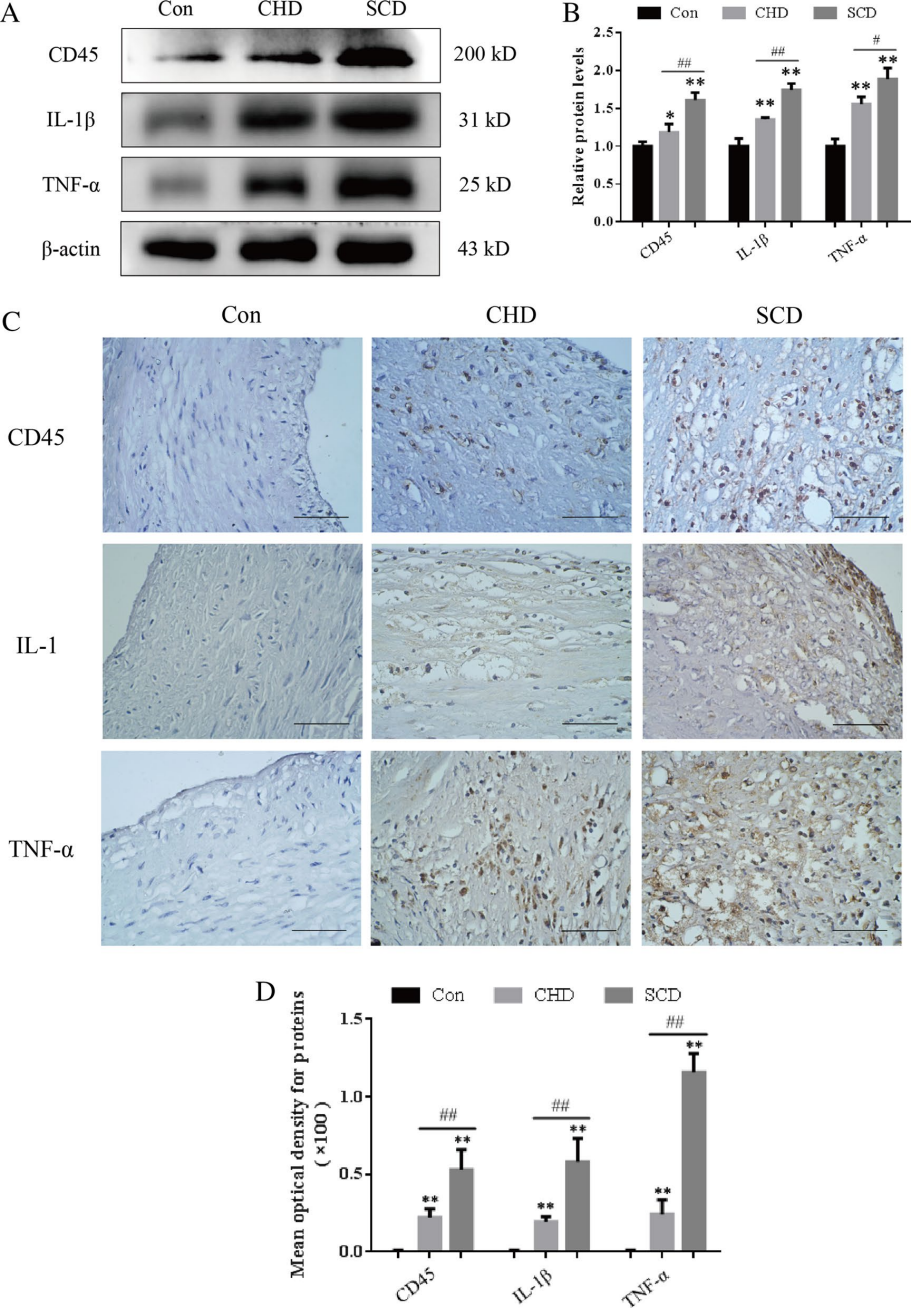
Protein expression levels in the coronary artery. **A, B** Representative Western blot images and quantitative analysis of protein expression levels. **C, D** Representative immunohistochemical images and quantitative analysis of protein expression in each group (scale bar = 50 μm). All experiments were repeated at least three times. The data are expressed as the mean ± SD (n = 12 per group). *P < 0.05 versus the control group; **P < 0.01 versus the control group; ##*P* < 0.01 versus the CHD group

The Western blot results showed that the levels of CD45, IL-1β and TNF-α in the lesion group were higher than those in the control group, and the expression of proteins in the SCD group was higher than that in the CHD group (Fig. 3A, B, P < 0.05). The immunohistochemistry results also showed that there was no expression of inflammatory factors in the intima in the control group, but in the atherosclerotic lesions, CD45 was expressed on the membrane of inflammatory cells in the shoulder area and the bottom of the lesions. IL-1β and TNF-α were mainly expressed in the cytoplasm and nuclei of foam cells around the atherosclerotic lesions, as indicated by yellow or brown staining (Fig. 3C). The mean density was measured by IPP6.0 and showed that positive expression in the lesion group was significantly higher than that in the control group, while positive expression in the SCD group was significantly higher than that in the CHD group, and the difference was statistically significant (Fig. 3D, P < 0.01).

### Expression of VCAM1 and MMP-9 in the coronary artery

**Fig. 4:**
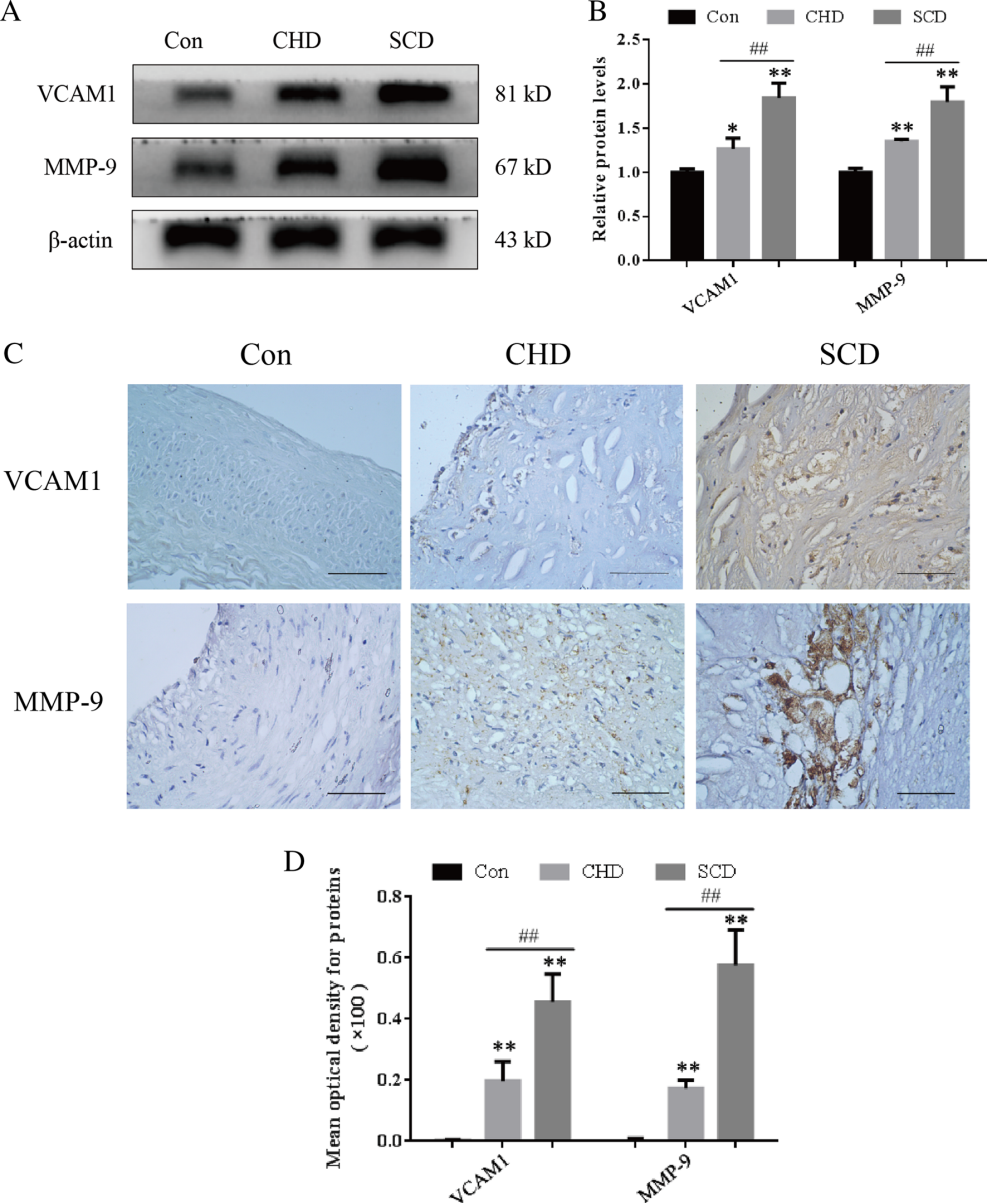
Protein expression levels in the coronary artery. **A, B** Representative Western blot images and quantitative analysis of protein expression levels. **C, D** Representative immunohistochemical images and quantitative analysis of protein expression in each group (scale bar = 50 μm). All experiments were repeated at least three times. The data are expressed as the mean ± SD (n = 12 per group). *P < 0.05 versus the control group; **P < 0.01 versus the control group; ##*P* < 0.01 versus the CHD group

The Western blot results showed that the expression of VCAM1 and MMP-9 in the lesion group was higher than that in the control group, and the expression of proteins in the SCD group was higher than that in the CHD group (Fig. 4A, B, *P* < 0.05). The immunohistochemistry results showed that there was no positive expression of VCAM1 or MMP-9 in the control group, but VCAM1 expression was found in vascular endothelial cells and subendothelial foam cells in both the CHD and SCD groups, while MMP-9 was mainly expressed in the cytoplasm of inflammatory cells in the shoulder area and bottom of the lesion, as indicated by yellow or brown staining of some nuclei (Fig. 4C). The optical density was measured by IPP6.0 and showed that positive expression in the lesion group was significantly higher than that in the control group, while positive expression in the SCD group was significantly higher than that in the CHD group, and the difference was statistically significant (Fig. 4D, *P* < 0.01).

### Correlation analysis of ATF3 protein expression in AS lesions and the structural indexes of the lesions

**Fig. 5:**
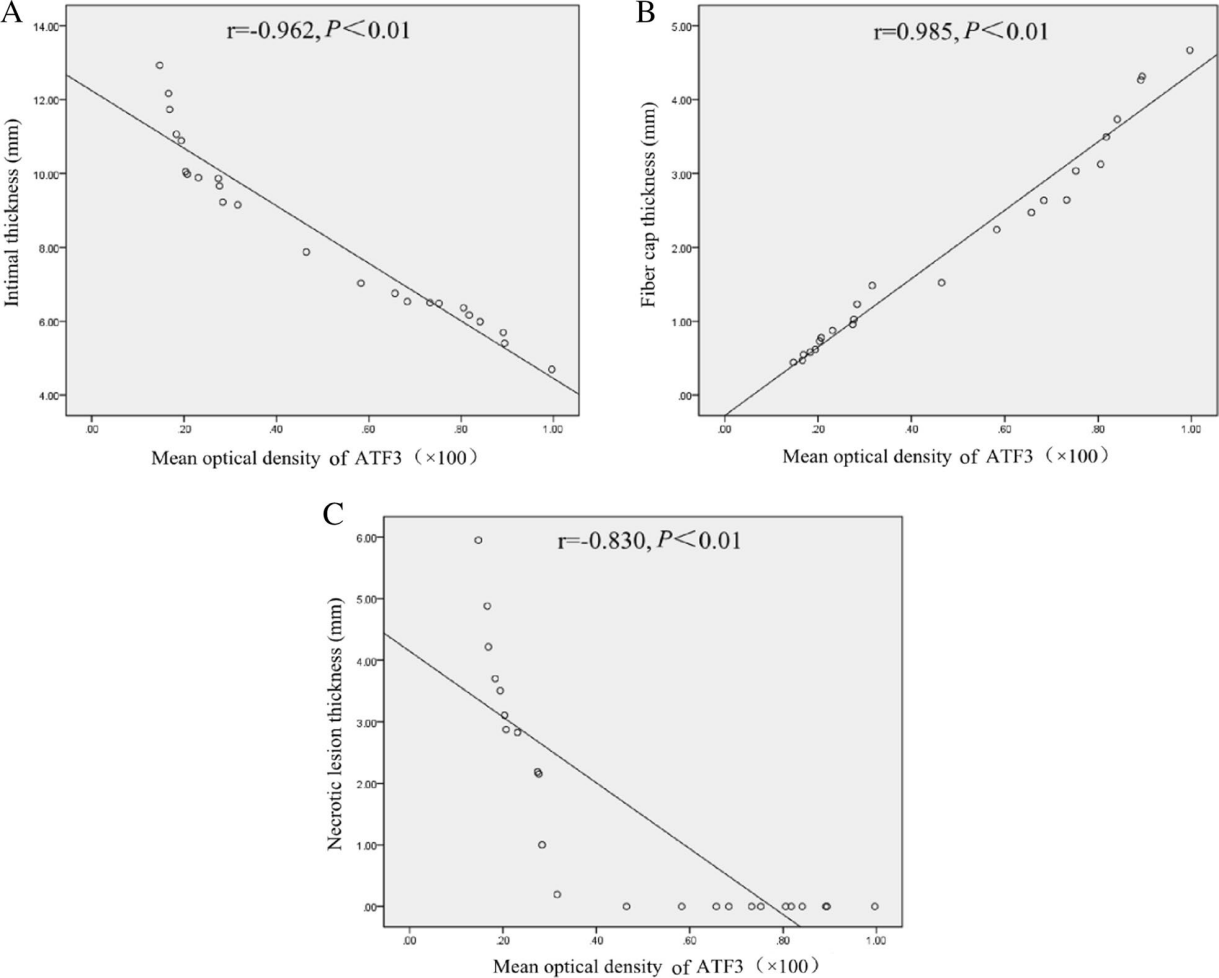
Correlations between the protein level of ATF3 and the structural indexes of the lesion in the lesion group. **A–C** Intimal thickness, fibrous cap thickness, and necrotic lesion thickness. r: Spearman’s correlation coefficient. *P* < 0.01 was considered to indicate a significant correlation

The correlations between the protein level of ATF3 in AS lesions and coronary intimal thickness, necrotic lesion thickness, fibre cap thickness and degree of lumen stenosis were analysed. The results showed that the protein level of ATF3 in atherosclerotic lesions was negatively correlated with coronary intimal thickness and necrotic lesion thickness (*P* < 0.01, Fig. 5A/C) and positively correlated with fibrous cap thickness (*P* < 0.01, Fig. 5B) but did not correlate with the degree of lumen stenosis (*P* > 0.05).

### Correlation analysis of ATF3 protein expression and inflammation in AS lesions

**Fig. 6:**
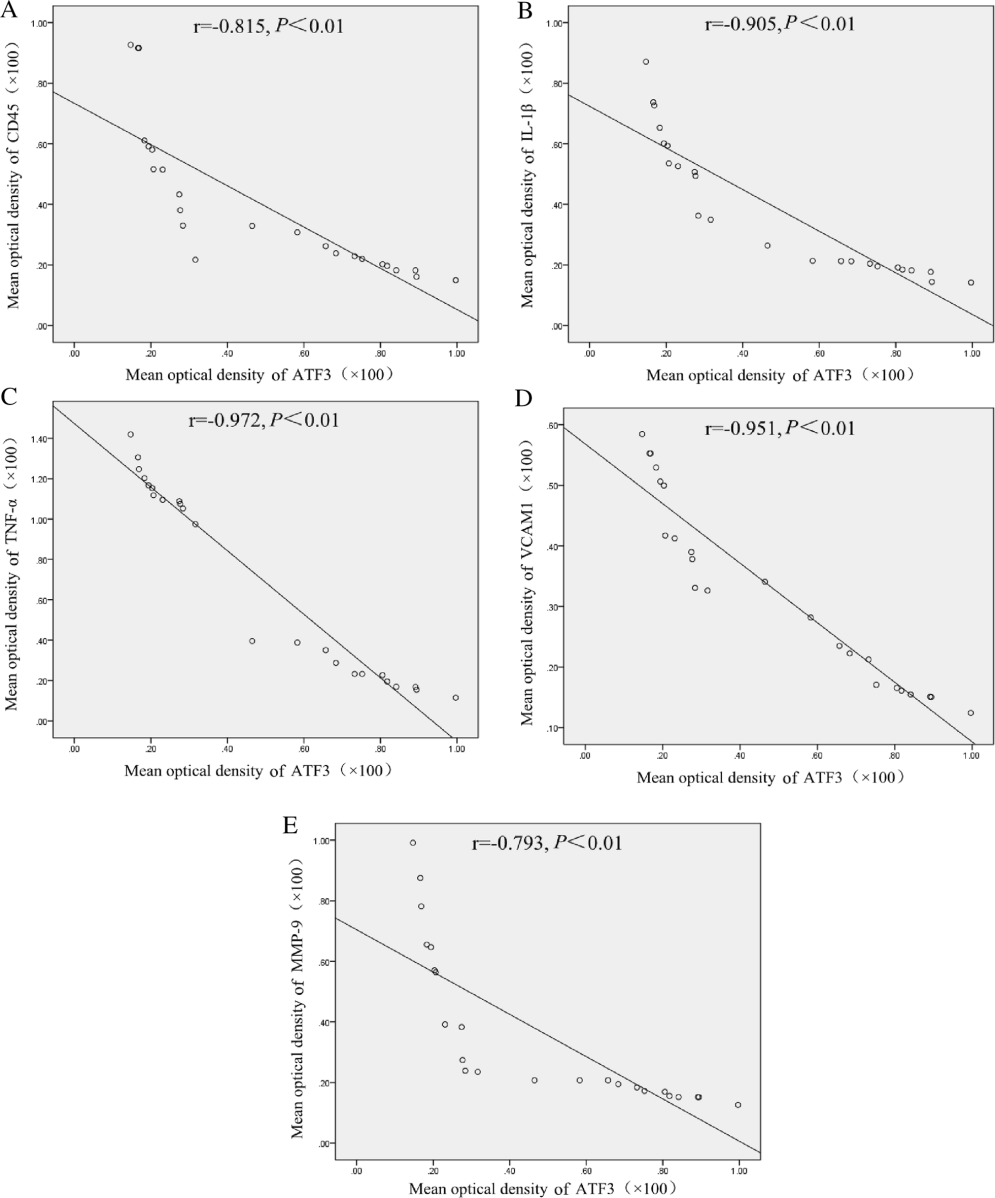
The correlation between ATF3 protein levels and inflammatory factors in the lesion group. r: Spearman’s correlation coefficient. **A–E** CD45, IL-1β, TNF-α and VCAM1, MMP-9. *P* < 0.01 was considered to indicate a significant correlation

The correlations between the protein level of ATF3 in atherosclerotic plaques and the inflammatory factors CD45, IL-1β, TNF-α, MMP-9 and VCAM1showed that ATF3 was negatively correlated with these factors (*P* < 0.01, Fig. 6).

## Discussion

The occurrence of sudden death due to CHD is closely related to the structural stability of atherosclerotic plaques [14, 15], but the factors affecting the stability of atherosclerotic plaques are still unclear. Research has shown that inflammation is a key factor that affects plaque stability [16]. In the pathogenesis of atherosclerosis, a variety of white blood cells, including monocytes, deposit under the intima and take up lipoproteins such as low-density lipoprotein and very-low-density lipoprotein to form foam cells [17]. They induce a serious inflammatory cascade, leading to vascular stenosis, intraplaque haemorrhage, plaque rupture and other secondary lesions, which can cause sudden death (Additional file 2).

Plaque stability is closely related to the onset of coronary heart disease and sudden death, and a consensus has gradually been reached that inflammation is a key factor in plaque. CD45 is expressed on the membranes of all inflammatory cells and is considered to be a marker of inflammation [18]. Both IL-1β and TNF-α are proinflammatory factors produced by inflammatory cells that mainly mediate the human immune and inflammatory responses [19–21]. Studies have shown that inflammation plays an important role in the occurrence and development of atherosclerosis. Activated leukocytes can adhere and accumulate in the vascular endothelium. And continue to release a variety of enzymes, cytokines and growth factors, resulting in persistent damage to blood vessels [22, 23]. Targeted inhibition of inflammatory cytokines such as IL-1β and TNF-α has gradually become a new therapeutic strategy against atherosclerotic vascular diseases [21, 24, 25]. Our study shows that the expression levels of CD45, IL-1β and TNF-α are significantly increased in human coronary atherosclerotic lesions, which indicates that inflammation plays an important role in atherosclerosis. Our study showed that the expression of CD45, IL-1β and TNF-α in atherosclerotic lesions in the SCD group was higher than that in the CHD group, suggesting that exacerbation of the inflammatory response was closely related to plaque instability.

Endothelial dysfunction is considered an early indicator of atherosclerosis and is characterized by the overexpression of adhesion molecules, including intercellular adhesion molecule-1 (ICAM-1) and vascular cell adhesion molecule-1 (VCAM-1) [26]. VCAM-1 mediates the rolling and adhesion of leukocytes, recruits leukocytes from the blood, and promotes monocytes to enter the vascular intima and express a variety of inflammatory cytokines after activation. The upregulated expression of VCAM-1 is considered to be a risk factor for plaque instability and myocardial infarction [27]. In late atherosclerotic lesions, the expression of MMP-9 is increased and can induce plaque destruction [28]. Volkov et al. [29] also confirmed that the level of MMP-9 was significantly increased in unstable fatty plaques, necrotic plaques and inflammatory erosive plaques. These studies suggest that VCAM-1 and MMP-9 play important early warning roles in unstable plaques. In our study, the expression of VCAM-1 and MMP-9 was increased in endothelial and subendothelial foam cells in the atherosclerotic group, especially in the SCD group, and the expression levels of VCAM-1 and MMP-9 in the atherosclerotic group were significantly higher than those in the CHD group, further suggesting that the increased expression of VCAM-1 and MMP-9 in the lesion could promote monocytes and other inflammatory cells to adhere to the endothelium, become activated and release a large number of inflammatory cytokines. Promoting the degradation of collagen in plaques leads to a decrease in plaque stability and the occurrence of sudden death.

Gold et al. showed that ATF3 was the key factor in lipid metabolism and the inflammation pathway in these cells that prevented atherosclerosis by inhibiting the formation of liposomes induced by 25-hydroxy [30]. By analysing the atherosclerotic data set from the NCBI-Gene Expression Omnibus database, it was found that the expression level of ATF3 in macrophages in ruptured atherosclerotic plaques was lower than that in stable atherosclerotic plaques, and it was further confirmed in animal experiments that a lack of ATF3 and an increase in macrophages may be risk factors for atherosclerotic plaque rupture [31]. The results of the present study showed that the expression level of ATF3 increased significantly in human coronary atherosclerotic plaques but decreased in the SCD group. By analysing the correlation between ATF3 expression and coronary artery structural indexes, it was found that ATF3 negatively correlated with coronary artery intimal thickness, necrotic core thickness and organ stenosis and positively correlated with fibrous cap thickness, intimal and dead focus thickening and fibre thickening. This finding indicates suggests that the expression of ATF3 may be related to the progression and stability of atherosclerotic plaques.

Malpass et al. [32] analysed the differential gene expression of human microvascular endothelial cells (HMEC-1) induced by cisplatin using the gene chip dataset GSE62523 and found that both ATF3 and VCAM1 may be key genes associated with endothelial injury. High-density lipoprotein (HDL) exerts protects the vasculature by promoting the activation of transcription factors (such as ATF3), resulting in downregulation of the inflammatory response induced by Toll-like receptors (TLRs) [33]. Targeted knockout of the ATF3 gene exacerbated cerebral ischaemia–reperfusion injury, significantly increased the expression of VCAM1 and MMP-9 [34], induced VSMC apoptosis and regulated the survival rates of vascular smooth muscle cells [35]. We analysed the correlation between ATF3, VCAM1 and MMP-9, and the results showed that there was a negative correlation between VCAM-1 and MMP-9. The important early warning role of VCAM1 and MMP-9 in unstable plaques further suggests that the occurrence of unstable atherosclerotic plaques may be related to the decrease in ATF3 levels. Related studies have shown that under the stimulation of various stress signals, ATF3 transcription activates many pathways, including NF-κB, MERK, JAK/STAT, and p38 [36]. The NF-κB signalling pathway plays a key role in regulating the inflammatory response in atherosclerosis. Phosphorylated NF-κB promotes the expression of many inflammatory genes, including VCAM-1, MCP-1, TNF-α, IL-1β and IL-6 [34]. The indirect interaction between ATF3 and NF-κB has been confirmed in lipopolysaccharide-activated macrophages and a renal ischaemia–reperfusion model [37]. Some studies have shown that ATF3 inhibits inflammation by directly interacting with the p65 subunit of NF-κB [38]. Therefore, we hypothesize that ATF3 may affect the stability of atherosclerotic plaques by regulating the inflammatory response.

## Conclusions

In summary, our study shows that in atherosclerosis, the degree of inflammatory reaction in plaques is related to plaque stability, the expression of ATF3 may be related to the progression and stability of atherosclerotic plaques, and may affect the structural stability of atherosclerotic plaques by regulating the inflammatory response in atherosclerotic plaques. This study was directly detected in human coronary artery, which made up for the deficiency of animal and cell experiments. However, all the cases come from the autopsy case database of the Forensic Judicial Expertise Centre of Guizhou Medical University. There is a problem that the collection of basic data is incomplete, such as the deceased's illness, medication and other information that may affect the experimental results can not be collected.

The current evidence shows that ATF3 can regulate cell dysfunction, such as apoptosis and inflammation, but whether ATF3 is a mediating factor or an inhibitory factor is still unclear, and the specific mechanism of ATF3 in atherosclerosis is still unclear. Therefore, we will use animal and cell models to further verify whether the deletion or over expression of ATF3 depends on the NF-κB signalling pathway to regulate the inflammatory response in atherosclerosis.

## Supplementary Information

**Additional file 1**. Basic case information.

**Additional file 2**. Protein bands primitive map.

## Data Availability

The datasets generated and analysed during the current study are not publicly available due the principle of funding confidentiality but are available from the corresponding author upon reasonable request.

## Abbreviations

ATF3: Activating transcription factor 3
CHD: Coronary heart disease
SCD: Sudden cardiac death
AS: Atherosclerosis
CD45: Leukocyte common antigen
IL-1β: Lnterleukin-1β
TNF-α: Tumor necrosis factor α
MMP-9: Matrix metalloproteinase-9
VCAM1: Vascular cell adhesion molecule 1
