# Supplementary figures and images for: Research on the correlation between activating transcription factor 3 expression in the human coronary artery and atherosclerotic plaque stability
Source: BMC Cardiovasc Disord. 2021 Jul 28;21:356. doi: 10.1186/s12872-021-02161-9 (PMC8317287; doi:10.1186/s12872-021-02161-9)

Fig 2. A

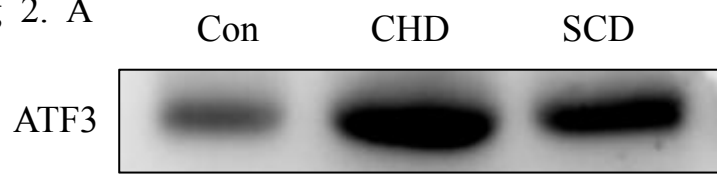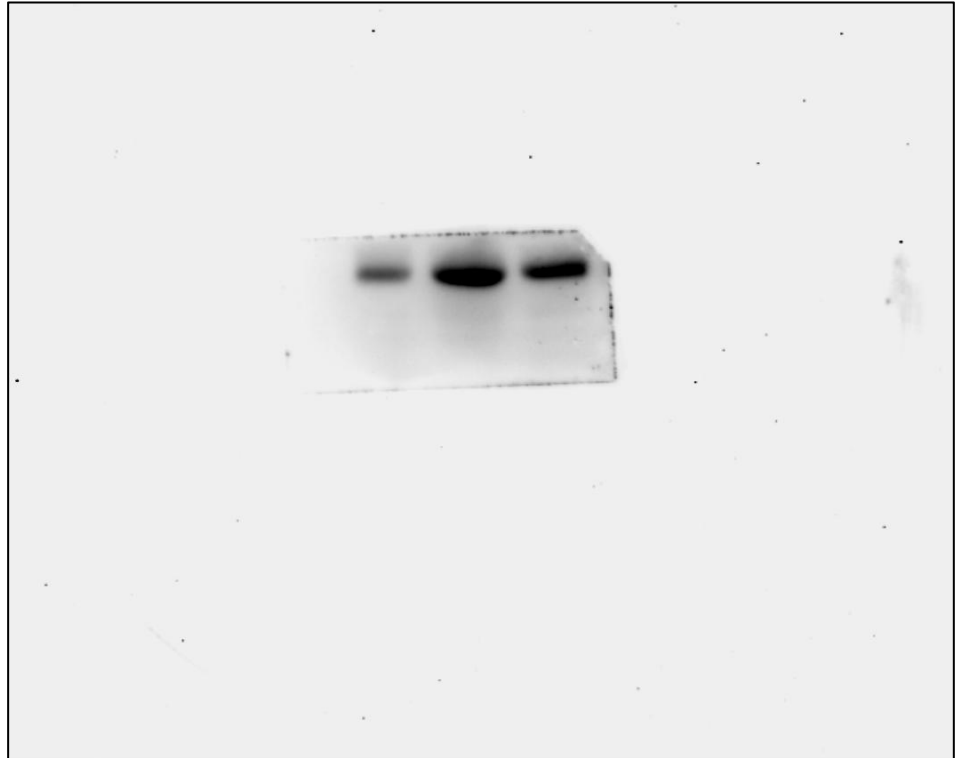

Fig 2. A

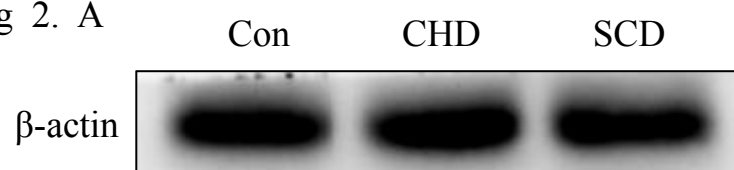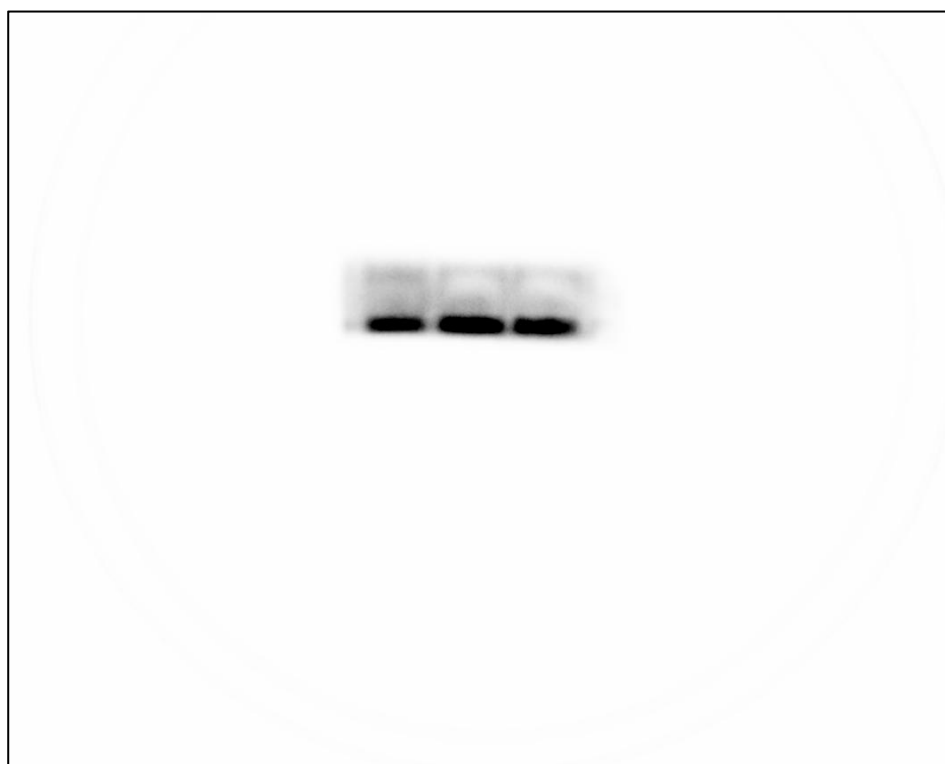

Fig 3. A

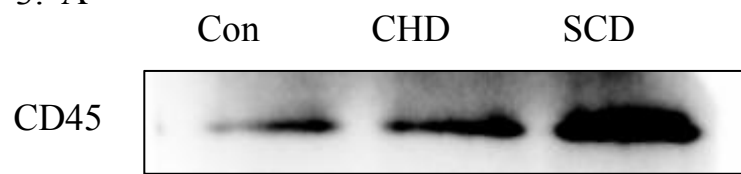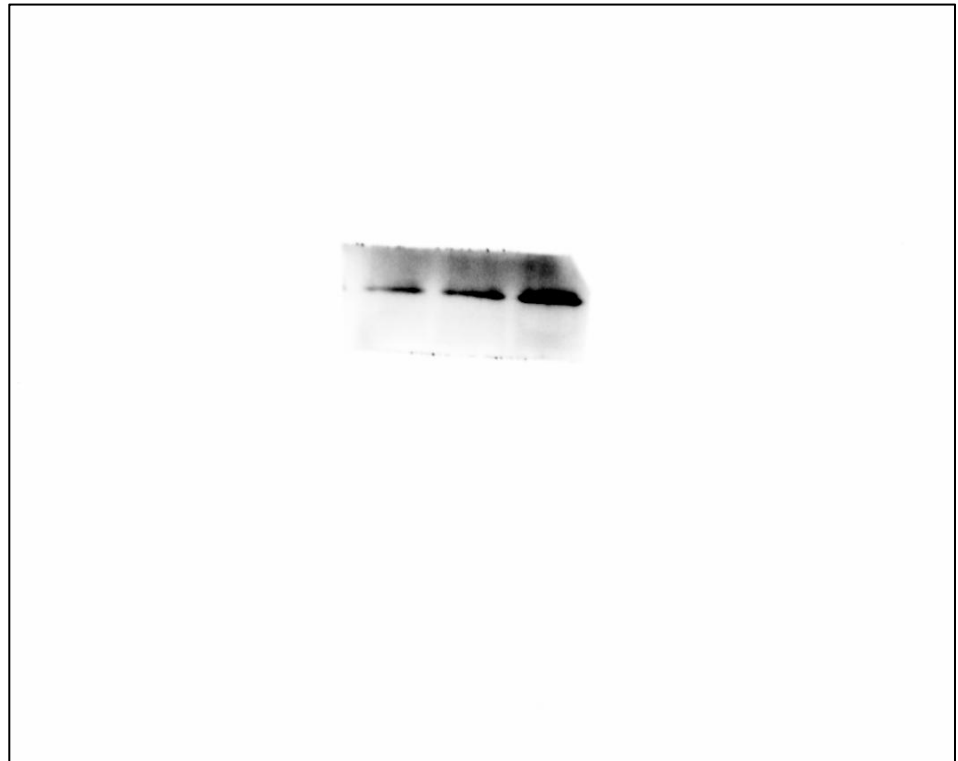

Fig 3. A

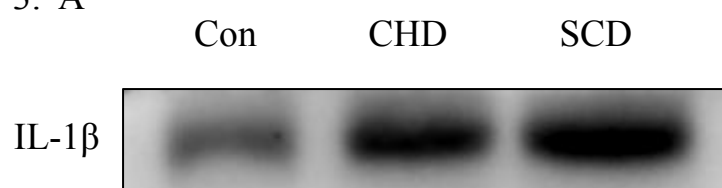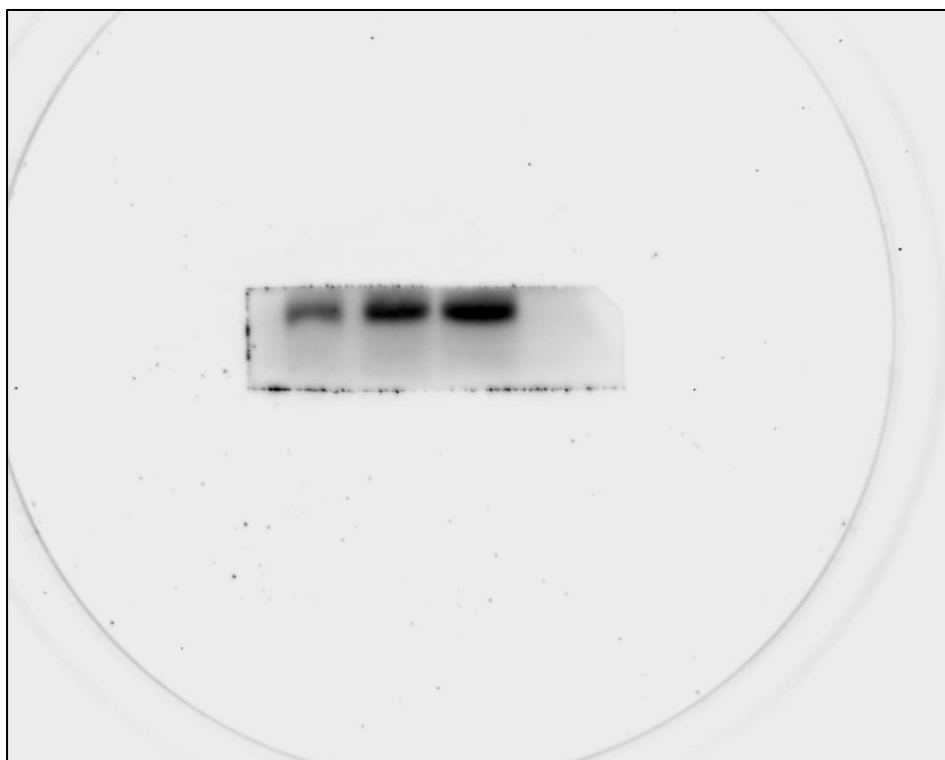

Fig 3. A

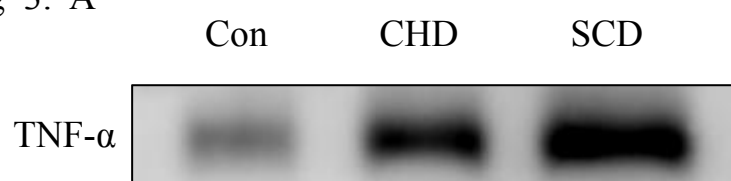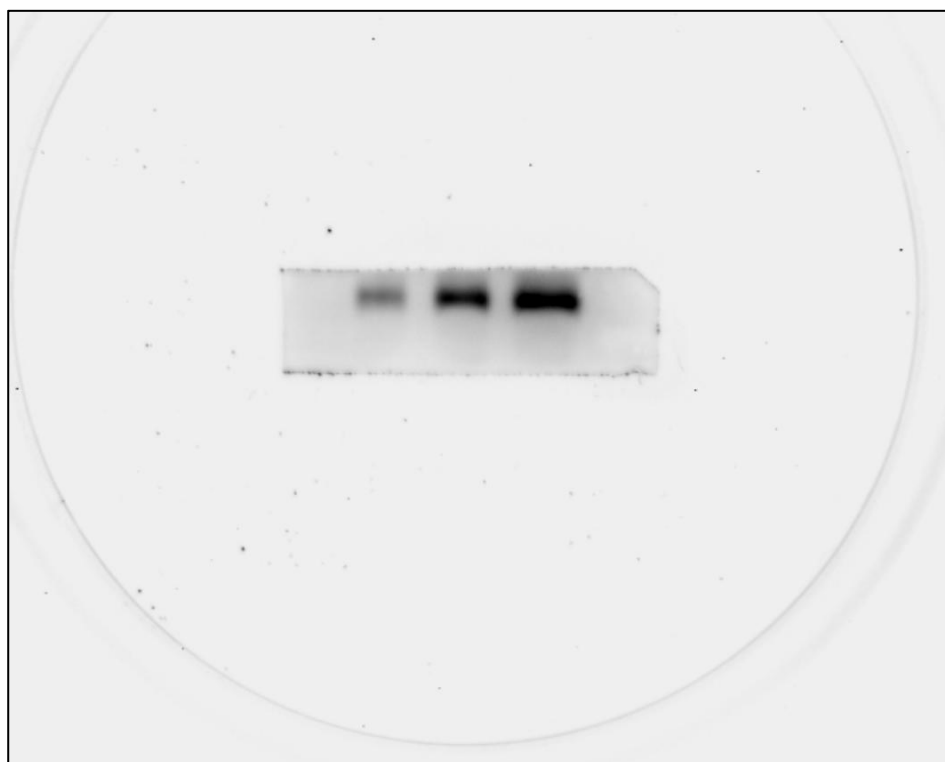

Fig 3. A

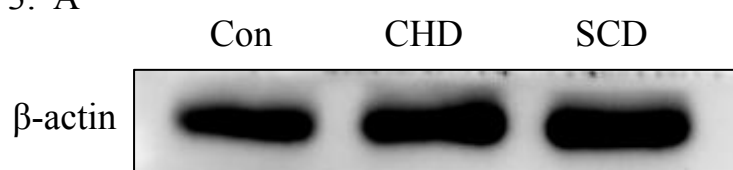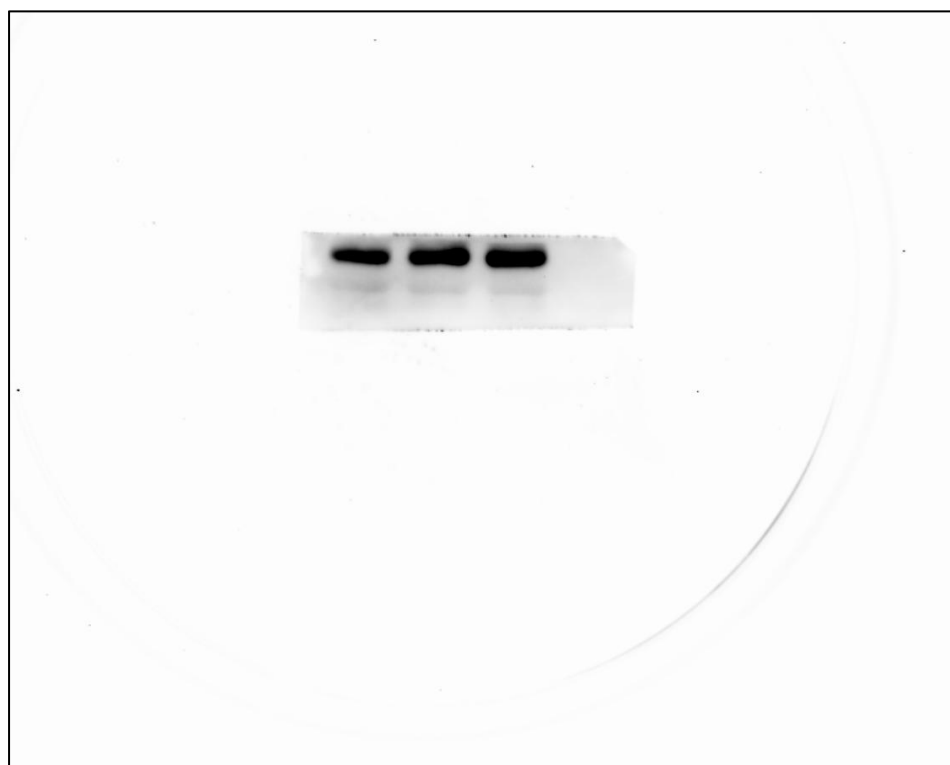

Fig 4. A

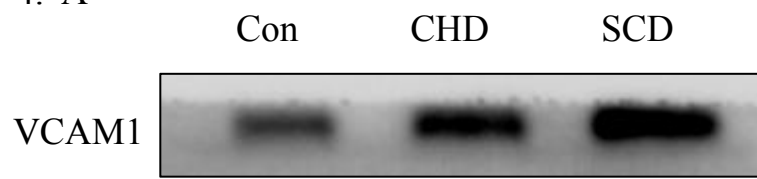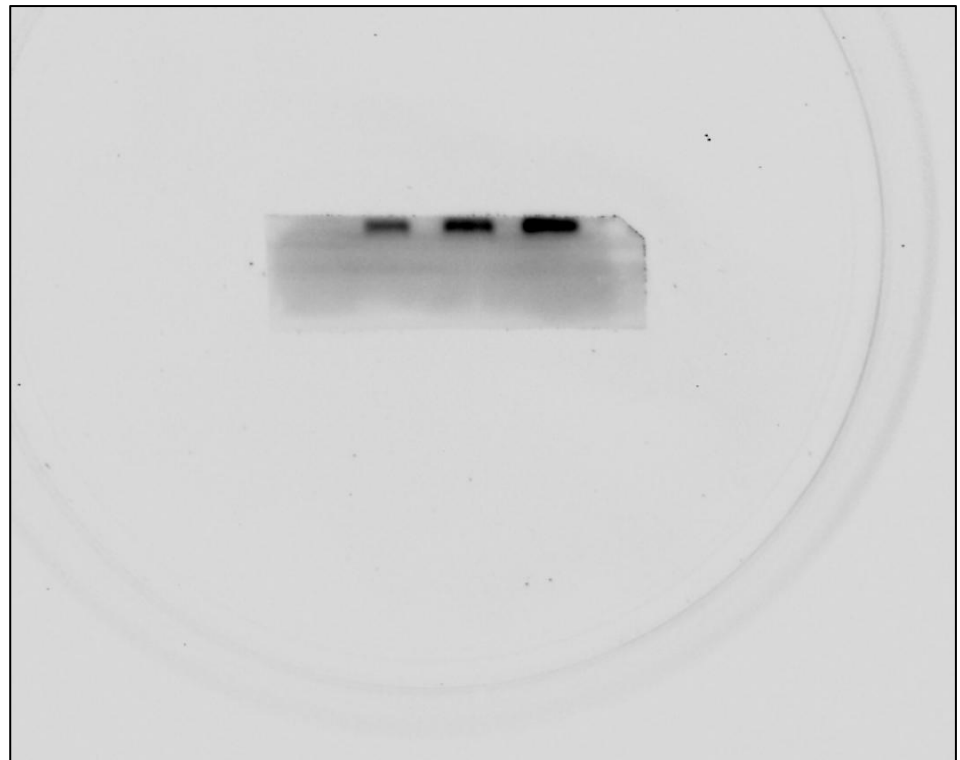

Fig 4. A

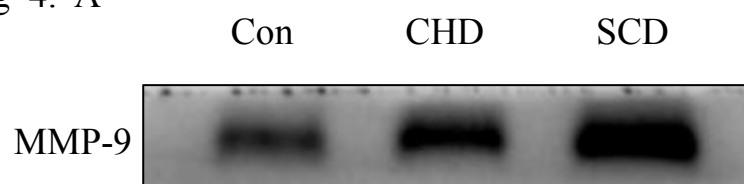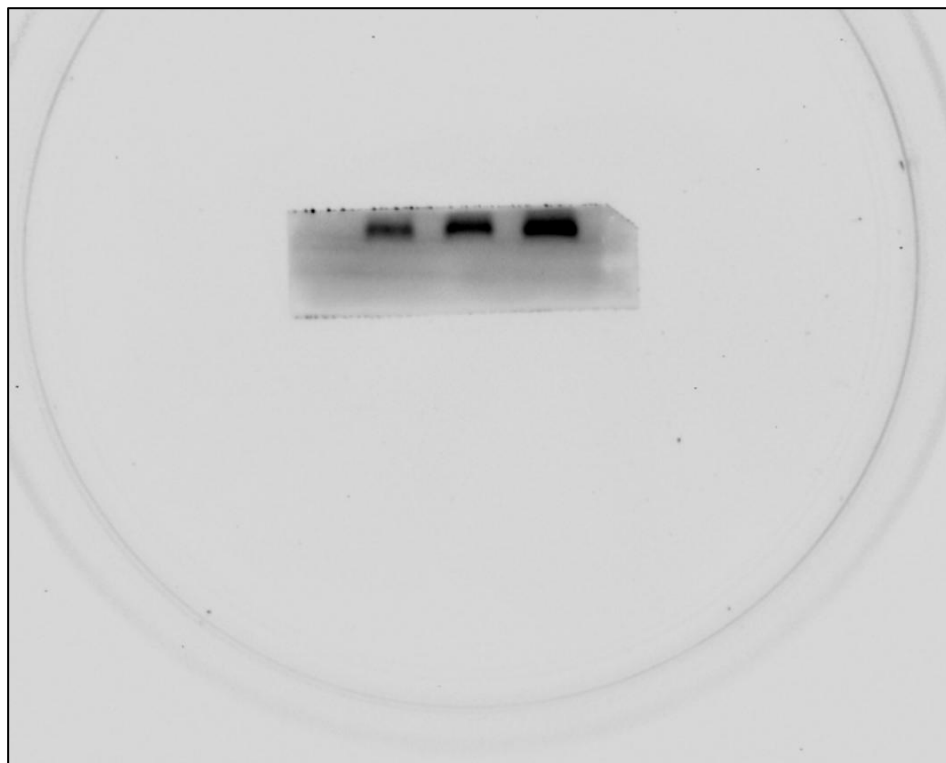

Fig 4. A

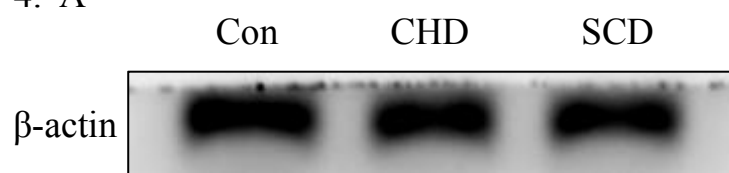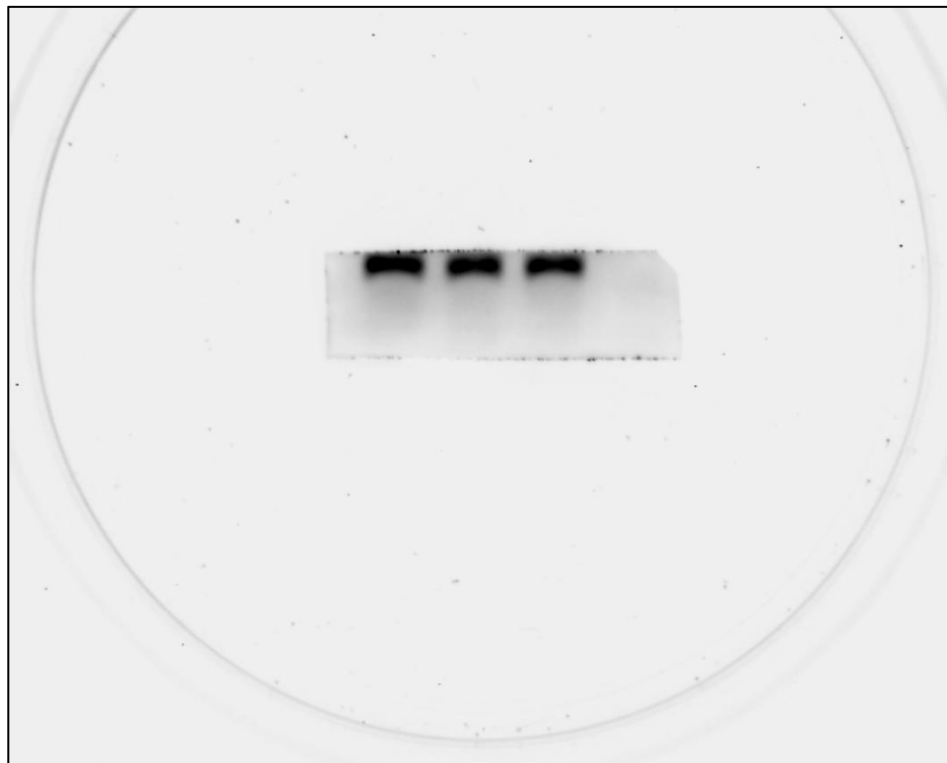

Supplement: Supplementary file 2 — Additional file 2. Protein bands primitive map. [file 12872_2021_2161_MOESM2_ESM.pdf]
